# Supplementary material for: Fh15 Reduces Colonic Inflammation and Leukocyte Infiltration in a Dextran Sulfate Sodium-Induced Ulcerative Colitis Mouse Model
Source: Cells. 2025 May 29;14(11):799. doi: 10.3390/cells14110799 (PMC12153920; doi:10.3390/cells14110799)
Supplement: Supplementary file 1 [file cells-14-00799-s001.zip › cells-3648544-supplementary.pdf]

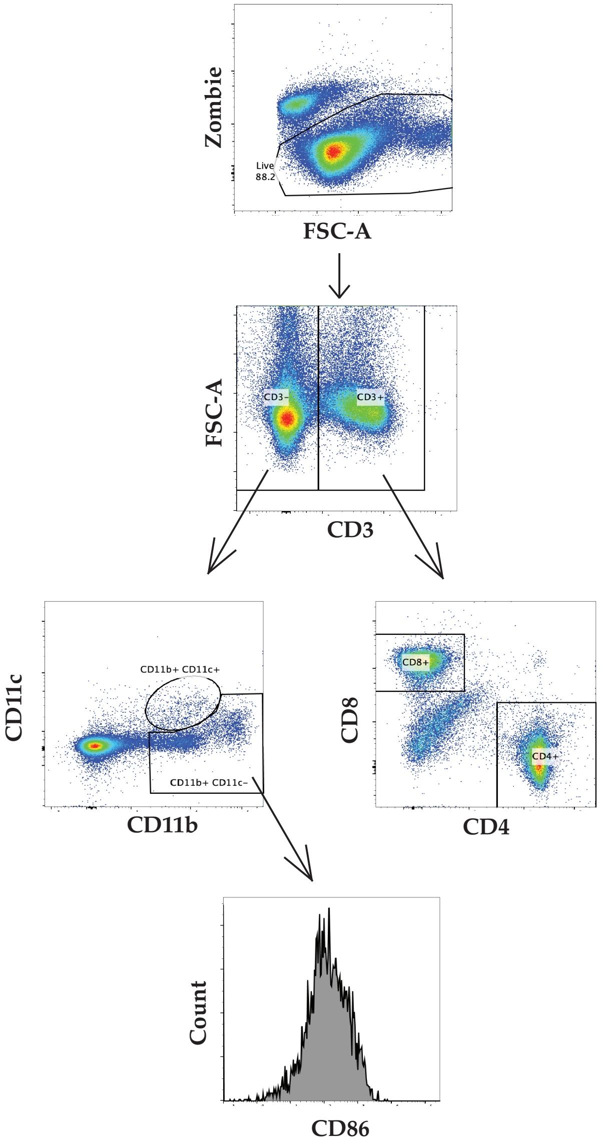

Figure S1. Schematic representation of the gating strategy for flow cytometry. Spleen lymphocytes were isolated, homogenized and single-cell suspensions of 5×10^5 cells per mouse were stained using a 10-color antibody panel for multiparametric flow cytometric analysis. Cells were incubated with an antibody cocktail containing Fc-block, live/dead Aqua Zoombie and then stained with antibodies against CD3, CD4, CD8, CD11b, CD11c, and CD86 (all from BD Biosciences, San Jose, CA, USA).

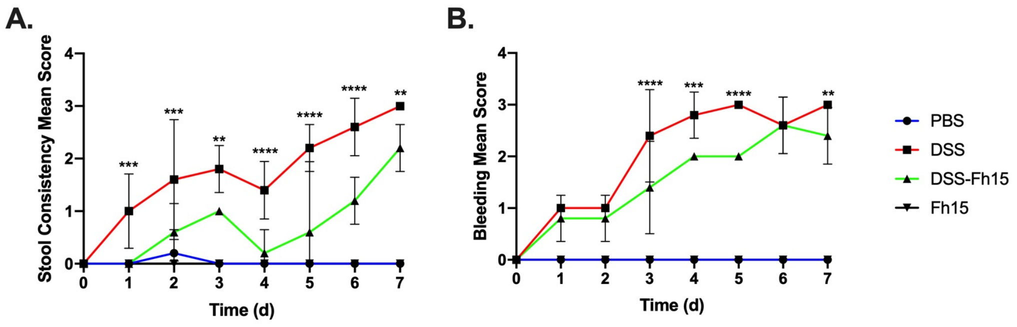

Figure S2. Stool consistency and bleeding is significantly reduced in DSS-induced UC mice that received Fh15 treatment. (**A**) Stool consistency and (**B**) bleeding of male C57BL5 mice treated with Fh15 on day 1, day 3, and day 5. Statistical significance between groups per day was assessed using two-way ANOVA with Dunnett's multiple comparison using DSS as reference group. ****p<0.0001, ***p<0.001, **p<0.01.

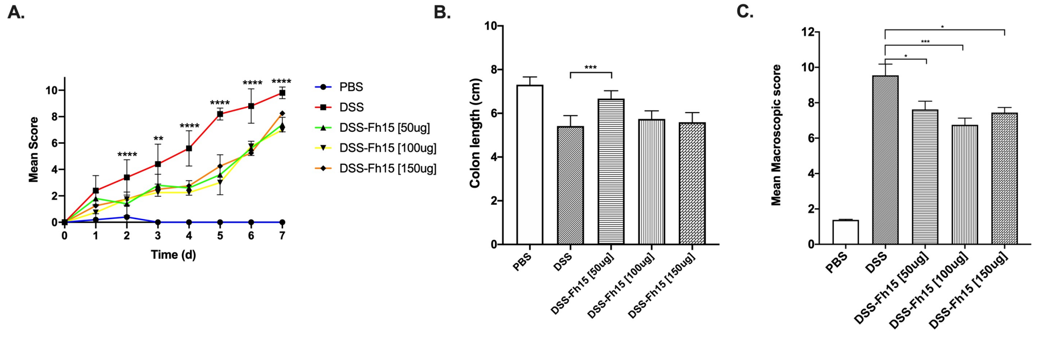

Figure S3. Fh15 dose-response, disease activity index, colon length, and macroscopic score index. **(A)** Disease activity index per day of C57BL/6 ulcerative colitis male mice treated with Fh15 at different concentrations (50μg, 100μg, and 150μg). **(B)** Males colon length after three doses of Fh15 at three different concentrations (50μg, 100μg, and 150μg). **(C)** Fh15 dose-response mean macroscopic score. Statistical significance for disease activity index between groups per day was evaluated using two-way ANOVA with Dunnett's multiple comparisons, using the DSS group as the reference, showing differences for Fh15 50μg only. For colon length and macroscopic score, significance between groups was determined using one-way ANOVA with Dunnett's multiple comparisons, also using the DSS group as the reference.*p<0.05, **p<0.01, ***p<0.001, ****p<0.0001.

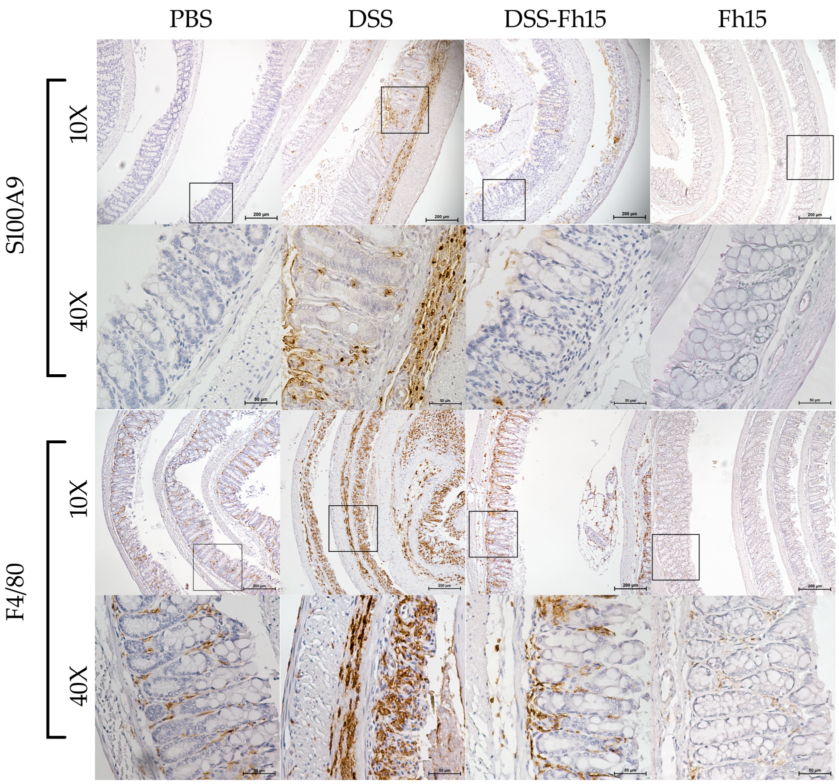

Figure S4. Treatment with Fh15 suppress the expression of S100A9 and the infiltration of macrophages in colonic tissue. Mice colon collected at day-7 of drinking 4% DSS-water were fixed in formalin, embeded in paraffin and cut into 4-µm-thick sections. Dewaxed colon sections were subjected to standard immunohistochemistry (IHC) using primary antibodies specific for S100 calcium binding A9 protein (S100A9) and macrophages (F4/80). Images shown representative results obtained for the negative control group (animals that only drank normal water and received i.p. injections with PBS), DSS-colitis (DSS), DSS-Fh15 (animals that drank DSS-water and received 3 i.p. injections with Fh15) and Fh15 (animals that drank normal water and received 3 i.p. injections with Fh15). The upper images set for each marker represent the colon section observed at 10X magnification. The square on each of these images represent the region that was magnified 40X.

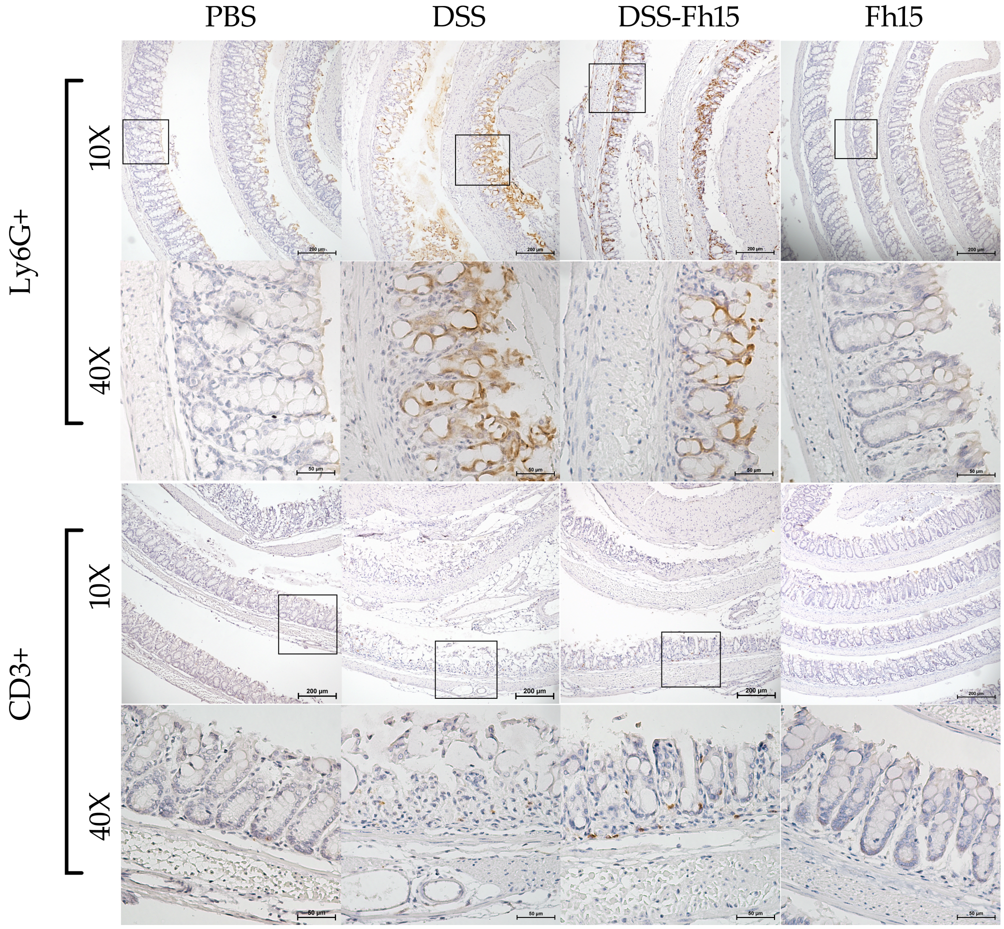

Figure S5. Treatment with Fh15 suppress the infiltration of neutrophils and T cells in colonic tissue. Mice colon collected at day-7 of drinking 4% DSS-water or normal water were fixed in formalin, embeded in paraffin and cut into 4-µm-thick sections. Dewaxed colon sections were subjected to standard immunohistochemistry (IHC) using primary antibodies specific for Ly6G (neutrophils) and CD3 (T cells). Images shown representative results obtained for the negative control group (animals that only drank normal water and received i.p. injections with PBS), DSS-colitis (DSS), DSS-Fh15 (animals that drank DSS-water and received 3 i.p. injections with Fh15) and Fh15 (animals that drank normal water and received 3 i.p. injections with Fh15). The upper images set for each marker represent the colon section observed at 10X magnification. The square on each of these images represent the region that was magnified 40X.

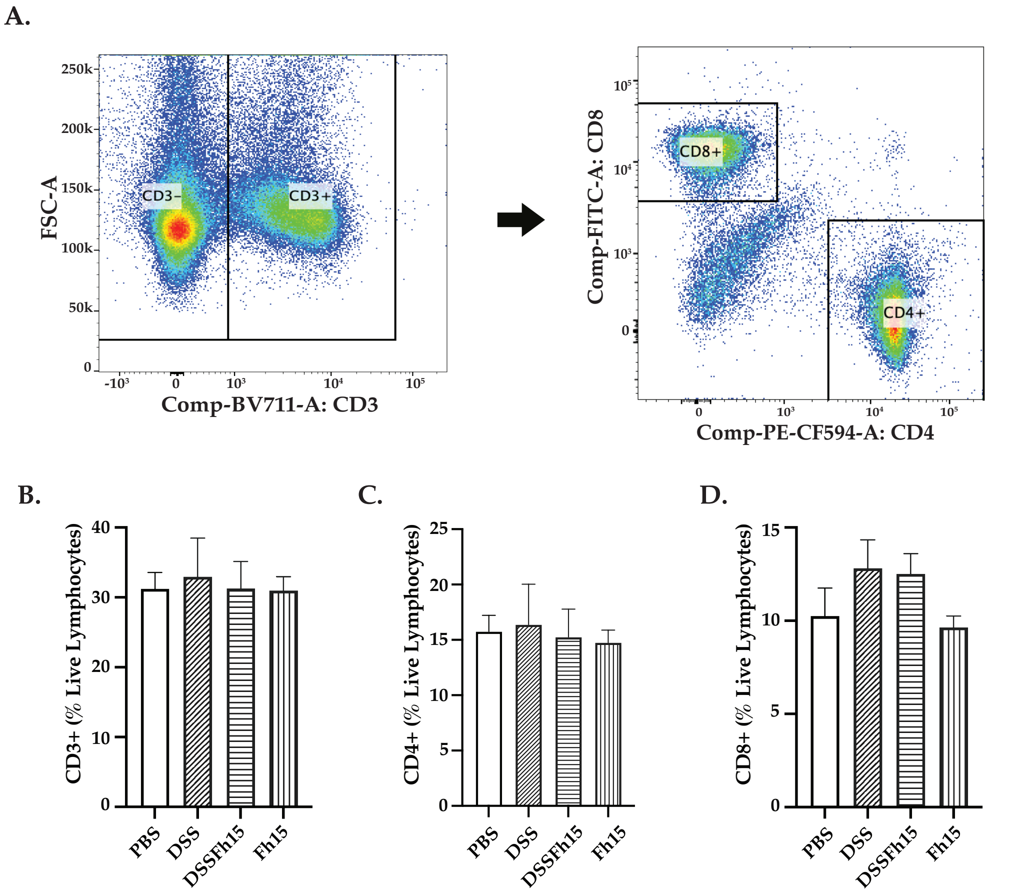

Figure S6. **Flow cytometry analysis for assessing spleen lymphocyte cell populations**. Spleen removed from each animal on the euthanasia day was cut into 3- to 5- mm pieces and homogenized. Prepared single-cell suspensions of 5×10^5 cells per mouse were stained with antibodies against CD3, CD4, and CD8. After fixation, cells were washed, resuspended in FACS buffer, and data were acquired using a 2-laser BD FACS Celesta flow cytometer and analyzed with FlowJo software version 10.6.2. **(A)** Live cell population that is CD3+ was gated and then separated by gating into CD8+ and CD4+ T cell populations. **(B)** Image represents percentages of live CD3+ T-lymphocytes **(C)** CD4+ T-lymphocytes and **(D)** CD8+ T-lymphocytes. No statistical differences were found between the percentage of live cells in the group exposed to DSS-water compared to naïve healthy controls (PBS) or treated with Fh15 that only drink normal water or between the DSS-group and those exposed to DSS that were treated with Fh15 for any of these markers.

A.

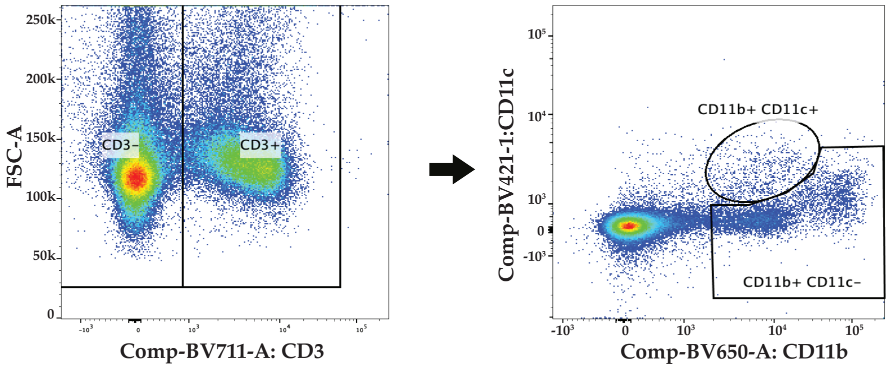

B.

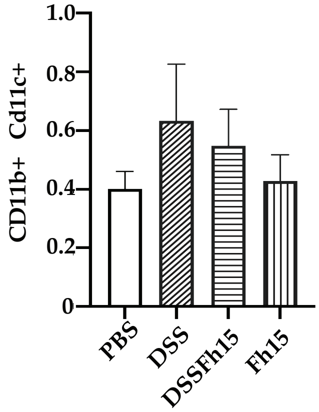

Figure S7. Flow cytometry analysis for assessing spleen dendritic cell populations. Prepared single-cell suspensions of 5 × 10^5 cells per mouse were stained with antibodies specific for CD11b+ and CD11c+. After fixation, cells were washed, resuspended in FACS buffer, and data were acquired using a 2-laser BD FACS Celesta flow cytometer and analyzed with FlowJo software version 10.6.2. **(A)** Live cell population that is CD3+ was separated by gating into CD11b+CD11c+ cell population, which represent the dendritic cells population. **(B)** No statistical differences in the number of DCs was found among the group exposed to DSS-water compared to naïve healthy controls (PBS), or treated with Fh15, which only drink normal water or between the DSS-group and the DSS-Fh15 group.

Table S1. Therapeutic effects of Fh15 in a DSS-induced ulcerative colitis mouse model, using a clinical scoring system to assess the disease activity index (DAI) on day 7.

Group	Mice #	Weight loss (%)	Stool consistency score	Presence of blood in stool score	DAI
**DSS**	1	28.33	3	3	10
	2	11.2	3	3	9
	3	22.55	3	3	10
	4	15.49	3	3	10
	5	19.38	3	3	10
	**Mean**	**19.39**	**3**	**3**	**9.8**
**DSS-Fh15**	1	10.45	3	2	8
	2	6	2	3	7
	3	12.59	2	3	8
	4	13.36	2	2	7
	5	10.72	2	2	7
	**Mean**	**10.62**	**2.2**	**2.4**	**7.4**

Table S2. Evaluation of Fh15 therapeutic efficacy in decreasing macroscopic colon damage and inflammation on day 7 after euthanasia.

Group	Mice #	Adhesion	Inflammed Length (cm)	Bowel thickness (mm)	Colon length	Hemorrhage	Fecal blood	Diarrhea	Total score
**DSS**	1	2	2.424	2.02	1	1	1	1	10.44
	2	2	1.619	1.87	1	1	1	1	9.49
	3	1	3.33	2.02	0	1	1	1	9.35
	4	1	1.675	1.67	0	1	1	1	7.35
	5	2	2.001	2.1	2	1	1	1	11.10
	**Mean**	**1.6**	**2.2098**	**1.936**	**0.8**	**1**	**1**	**1**	**9.546**
**DSS-Fh15**	1	1	0.803	1.9	1	1	1	1	7.70
	2	2	1.554	1.82	0	1	1	1	8.37
	3	1	1.762	1.76	1	1	1	1	8.52
	4	2	1.768	1.85	1	0	1	0	7.62
	5	1	1.939	1.96	0	0	1	0	5.90
	**Mean**	**1.4**	**1.5652**	**1.858**	**0.6**	**0.6**	**1**	**0.6**	**7.622**

Table S3. Average histopathological score with standard deviation per evaluated parameter by experimental group.

Group	Extent of inflammation	Infiltration neutrophils + lympho-histiocytes	Extent of crypt damage	Crypt abscesses	Sub-mucosal edema	Loss of goblet cells	Reactive epithelial hyperplasia
**PBS**	0	0.33 ± 0.5773	0	0	0	0	0
**DSS**	2	2	2.66 ± 0.5773	0.66 ± 0.5773	1.66 ± 0.5773	2.33 ± 0.5773	2 ± 1
**DSS-Fh15**	1.66 ± 0.5773	1.33 ± 0.5773	1.66 ± 0.5773	0	1.66 ± 0.5773	1	1.33 ± 0.5773
**Fh15**	0	0.5 ± 0.7071	0	0	0	0	0

49 Spadina Ave. Suite 200
Toronto ON M5V 2J1 Canada
www.biorender.com

Confirmation of Publication and Licensing Rights - Open Access

May 21st, 2025

Subscription Type: *Institution - Academic*
Agreement number: *GP28AND0FW*
Publisher Name: *Cells*

Figure Title: *DSS-induced ulcerative colitis and Fh15 treatment administration in C57BL/6 male mice*

Citation to Use: *Created in BioRender. Figueroa, M. (2025) https://BioRender.com/n0aacjy*

To whom this may concern,

This document ("Confirmation") hereby confirms that Science Suite Inc. dba BioRender ("BioRender") has granted the following BioRender user: María Del Mar Figueroa ("User") a BioRender Academic Publication License in accordance with BioRender's Terms of Service and Academic License Terms ("License Terms") to permit such User to do the following on the condition that all requirements in this Confirmation are met:

1) publish their Completed Graphics created in the BioRender Services containing both User Content and BioRender Content (as both are defined in the License Terms) in publications (journals, textbooks, websites, etc.); and
2) sublicense such Completed Graphics under "open access" publication sublicensing models such as CC-BY 4.0 and more restrictive models, so long as the conditions set forth herein are fully met.

Requirements of User:

1) All Completed Graphics to be published in any publication (journals, textbooks, websites, etc.) must be accompanied by the following citation either as a caption, footnote or reference for each figure that includes a Completed Graphic:
"Created in BioRender. Figueroa, M. (2025) https://BioRender.com/n0aacjy ".
2) All terms of the License Terms including all Prohibited Uses are fully complied with. E.g. For Academic License Users, no commercial uses (beyond publication in journals, textbooks or websites) are permitted without obtaining or switching to a BioRender Industry Plan.
3) A Reader (defined below) may request that the User allow their figure to be a public template for Readers to view, copy, and modify the figure. It is up to the User to determine what level of access to grant.

Open-Access Journal Readers:

Open-Access journal readers ("Reader") who wish to view and/or re-use a particular Completed Graphic in an Open-Access journal subject to CC-BY sublicensing may do so by clicking on the URL link in the

applicable citation for the subject Completed Graphic.

The re-use/modification options below are available after the Reader requests the User to adapt their figure as a BioRender template and the User has granted such access.

1) View-Only/Free Plan Use: A Reader who wishes to only view the Completed Graphic may do so in the BioRender Services as either a BioRender Free Plan user or simply as a viewer. By becoming a BioRender Free Plan user, the Reader may view, modify and re-use the Completed Graphic as permitted under BioRender's Basic License Terms (e.g. personal use only, no publishing or commercial use permitted).
2) Re-Use/Publish with No Modifications: For any re-use and re-publication of a Completed Graphic with no modification(s) to the Completed Graphic made by the Reader, a Reader may do so by citing the original author using the citation noted above with the Completed Graphic. The Reader must also comply with the underlying License Terms which apply to the Completed Graphic as noted above (e.g. no commercial use for Academic License).
3) Re-Use/Publish with Modifications: For any re-use and re-publication of a Completed Graphic with a modification(s) made by the Reader, the Reader may do so by becoming a BioRender user themselves under either an Academic or Industry Plan, citing the original author using the citation noted above with the Completed Graphic and complying with the applicable License Terms.

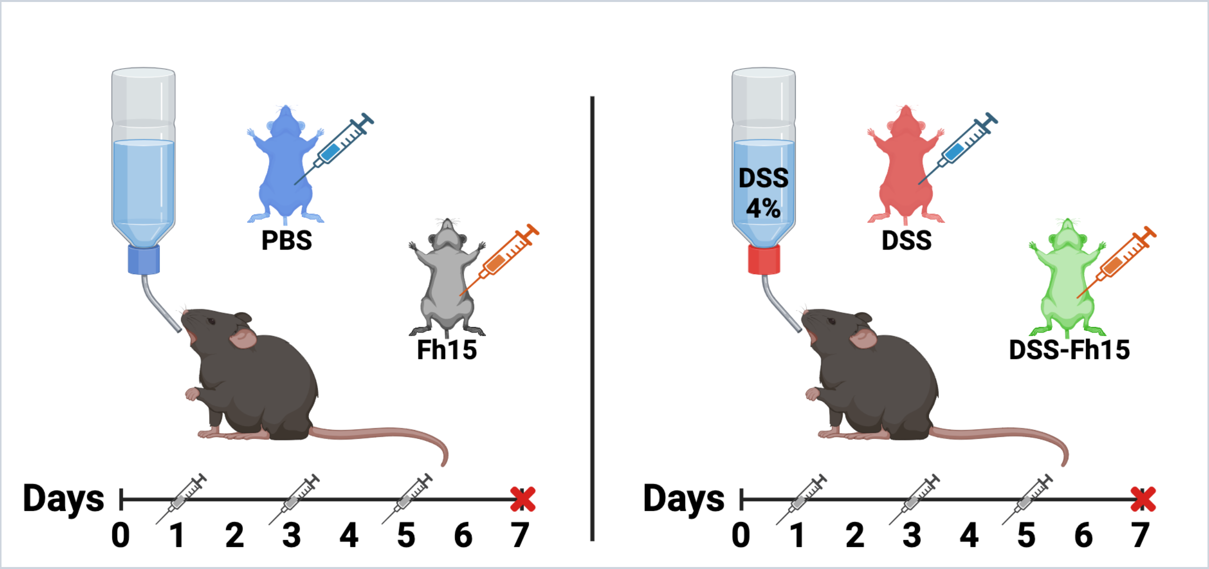

For any questions regarding this document, or other questions about publishing with BioRender, please refer to our BioRender Publication Guide, or contact BioRender Support at support@biorender.com.
